# A Review of Potential Exoskeletons for the Prevention of Work-Related Musculoskeletal Disorders in Agriculture

**DOI:** 10.3390/s24217026

**Published:** 2024-10-31

**Authors:** Sanura Dunu Arachchige, Lasitha Piyathilaka, Jung-Hoon Sul, D. M. G. Preethichandra

**Affiliations:** School of Engineering and Technology, Central Queensland University, Rockhampton, QLD 4701, Australia; l.piyathilaka@cqu.edu.au (L.P.); j.sul@cqu.edu.au (J.-H.S.)

**Keywords:** exoskeletons, Work-Related Musculoskeletal Disorders (WMSD), agriculture, assistive robots, ergonomics

## Abstract

Exoskeletons possess a high potential for assisting the human workforce while eliminating or reducing the risk of Work-Related Musculoskeletal Disorders (WMSDs). However, their usage in agricultural work, where there is a plethora of reported WMSD cases, seems limited. Since agricultural tasks are complex and performed in harsh environments, developing novel exoskeleton-based solutions could be challenging. However, commercial exoskeletons are already being used in various other industries, such as logistics, military, medicine, and manufacturing. Thus, it is expected that those existing exoskeleton solutions could be applied to agricultural tasks. Nevertheless, prior to implementation, assessing the feasibility, efficacy, and necessary modifications for these exoskeletons is imperative to supporting agricultural activities prone to WMSDs. In this review, prevalent exoskeletons documented in scientific literature are identified, and their potential relevance to agricultural tasks with elevated WMSD risks is evaluated. The review further highlights and deliberates on exoskeletons that could be applicable in an agricultural context. This comprehensive examination serves as a foundational step towards the conceptualization and development of exoskeleton-based approaches tailored explicitly for agricultural tasks.

## 1. Introduction

Work-Related Musculoskeletal Disorders (WMSDs) significantly affect the present labor force, especially in labor-intensive industries such as agriculture [1,2,3,4,5]. In previous research, numerous investigations on WMSDs related to agriculture have found the causes, effects, and a few solutions for injury prevention [1,2,6,7,8,9,10,11,12,13,14]. However, the outcomes of these studies are yet to be seen in the agricultural sector. Exoskeleton technology has been assisting human workers for more than a century and has a high potential for reducing or eliminating the risk of WMSDs. The first recorded (patented) exoskeleton was introduced in 1890 [15], and the field has consistently progressed since then to commercially available exoskeletons that are actively used in various industries [16,17,18,19,20,21,22,23,24,25,26,27,28]. Even though there is a handful of exoskeletons in experimental stages, which have a high potential for agricultural tasks [29,30,31,32], surprisingly, there are no industrial-level/real-world applications of exoskeletons in agriculture, where there is a high need for such solutions. It is evident that agricultural industries in developing countries, where small-scale farming is the dominant sector in agricultural production [33], rely heavily on human labor [11,33,34,35,36,37,38,39,40]. On the other hand, developed nations, which are the primary contributors to exoskeleton research, focus more on automation in their large-scale agriculture. Therefore, the trend of exoskeleton research has focused on other industries, such as military, manufacturing, and dependent-care industries, rather than the agricultural sector [16,17,18,19,20,21,22,23,24,25,26,27,28].

Since agricultural tasks involve complex motions and harsh environments, developing an exoskeleton specifically for the agricultural sector would be highly challenging. Hence, it would be prudent to prioritize an investigation into the feasibility of utilizing existing exoskeletons rather than embarking on the development of an entirely new design from the ground up. The first part of this study aims to explore exoskeletons used in various other industries that have the potential to be used in agricultural activities. It is expected that the introduction of exoskeleton technology to agriculture would reduce the WMSD risks involved in agriculture, making the industry efficient and safe. It is important to conduct a comprehensive review of both exoskeleton technologies (active and passive) and the specific needs of agricultural tasks, which is performed in this study. This critical review of the existing exoskeletons enables the evaluation of their effectiveness across various agricultural tasks.

This review will commence with an in-depth exploration of Work-Related Musculoskeletal Disorders (WMSDs). Then, the latest exoskeleton developments found in recent publications are discussed and summarized. Subsequently, the applicable exoskeletons to agriculture are sorted. Finally, challenges, considerations, and ethical factors are discussed.

### 1.1. Aim

The aim of this review is to identify and suggest available exoskeletons that have the potential to assist agricultural work in reducing or eliminating the risks of work-related musculoskeletal disorders.

### 1.2. Objectives

The objectives of this study are to,

1. Identify agricultural tasks that cause risks for WMSDs.
2. Identify different exoskeletons in various other industries, which are designed to support tasks similar to agricultural tasks with risks of WMSDs in agriculture.
3. Identify characteristics of exoskeletons that are suitable for agricultural tasks.

## 2. Materials and Methods

**Figure 1:**
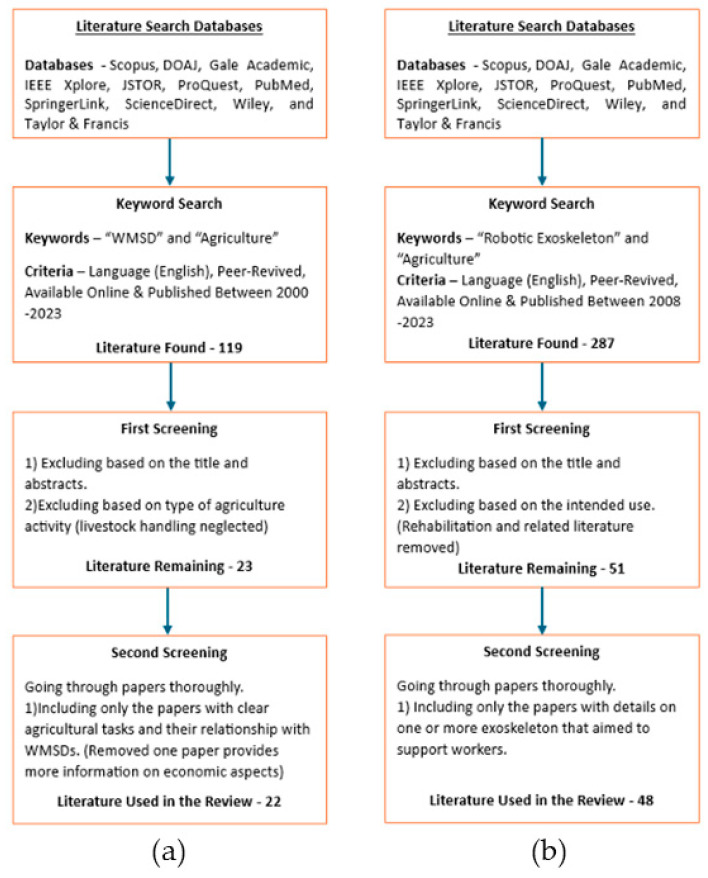
(**a**) WMSD-related literature selection criteria; (**b**) Exoskeleton-related literature selection criteria.

Literature searches were carried out in two categories. Initially, a search was performed to find literature related to work-related musculoskeletal disorders in various recognized databases, including DOAJ, Gale Academic, IEEE Xplore, JSTOR, ProQuest, PubMed, SpringerLink, Science Direct, Wiley, and Taylor & Francis. The keywords for this search were “WMSD” and “Agriculture”. This search criteria included the language (English), peer-reviewed status, publication years (2000–2023), and online availability. Initially, 119 papers were found, which were then screened based on their titles, abstracts, and relevance to agriculture. Then, they were narrowed down to 23 papers by the authors. A thorough reading of these 23 papers was conducted by the authors, who identified countries and regions where the WMSDs were reported, the reasons for WMSDs (activities and tasks), and current solutions. Finally, 22 papers were selected for citation, as illustrated in Figure 1a.

The same procedure was used to find literature related to exoskeletons. The aforementioned databases were searched using the keywords “robotic exoskeletons” and “agriculture”. A total of 287 articles written in English, peer-reviewed, online available, and published between 2008 and 2023 were found. The reason for selecting this 15-year period is based on the identified exoskeleton-related scientific literature production trends [34]) that were identified. After filtering based on their titles and abstracts, 51 papers remained. Following a thorough reading of these 51 papers by the authors to identify exoskeletons that are fabricated and at least tested in a laboratory environment and not specifically designed for rehabilitation, 48 papers were selected for citation in this review, as shown in Figure 1b.

## 3. WMSDs and Related Agricultural Tasks

WMSDs arise when muscles, tendons, nerves, or joints experience repeated stress and trauma over days, months, or years, leading to tissue damage and injury [35,36,37]. When human workers are involved in repetitive tasks, awkward postures, heavy lifting, prolonged static postures, and/or climbing (trees, ladders, walls, etc.) that strain their musculoskeletal system, they are susceptible to frequent work-related injuries. The aforementioned activities that put workers at risk of work-related injuries are commonly observed in labor-intensive fields such as manufacturing, agriculture, and logistics [38]. While WMSDs in the agricultural sector are very common [39,40,41,42,43], there have been only a handful of solutions proposed by previous research works to mitigate them [15,44]. The most prominent solution found in the literature for reducing the risks associated with agricultural tasks is correcting postures [3,4]. However, changing traditional practices and habits can be challenging and need wide-scale awareness programs. Therefore, novel solutions such as exoskeletons can be sought.

**Figure 2:**
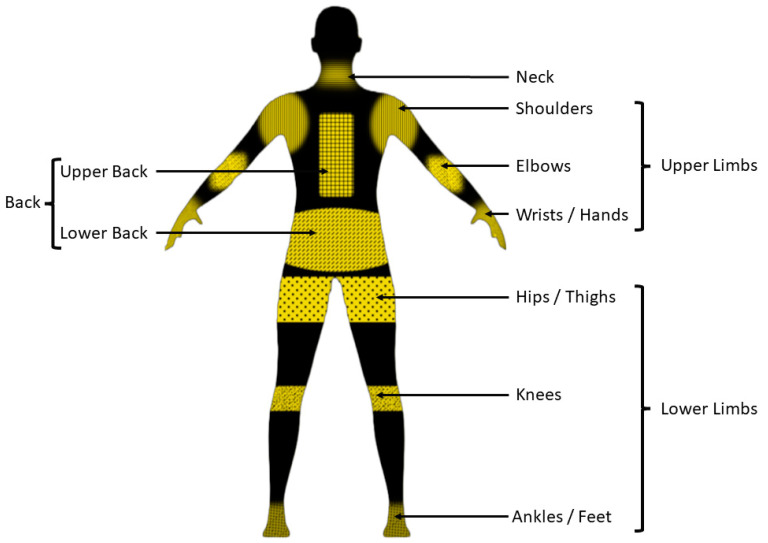
WMSD-occurring regions categorized anatomically.

WMSDs can cause both short-term and long-term damage to agricultural workers. In the short term, these will affect their performance and cause discomfort in both daily activities and agricultural tasks [45,46,47]. In the long term, these can cause devastating effects on workers’ bodies, causing them to be permanently injured/disabled and ultimately lose their jobs [37,48,49]. WMSDs can occur in various locations in a human body, either in a single location or multiple locations simultaneously [50]. Scientific literature depicts that the effects of WMSDs could be observed on regions such as the neck, shoulders, upper/lower back, hands/wrists, elbows, hips/thighs, knees, and feet/ankles, which can be identified by human anatomy, as shown in Figure 2. These WMSDs can be caused by a range of factors, including repetitive motions, awkward postures, prolonged work duration, handling heavy physical loads (lifting), bending, climbing, reaching, and twisting [1,2,7,11,12,14,46,50,51,52,53,54,55,56,57,58,59].

**Figure 3:**
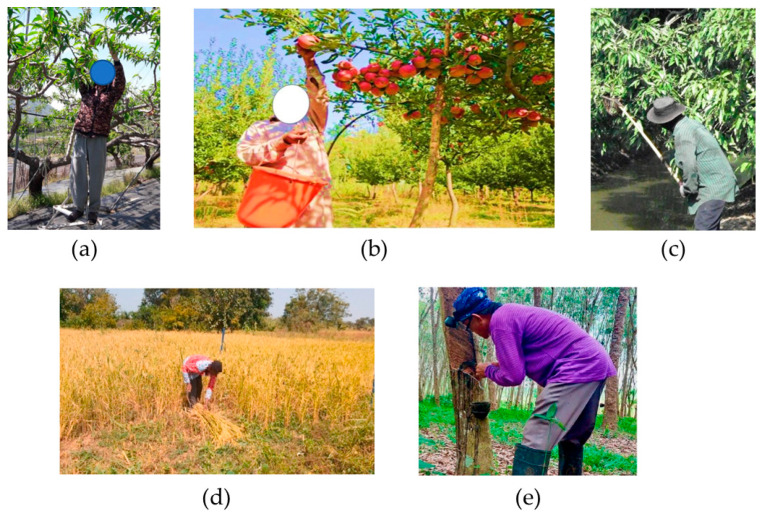
Typical agricultural tasks: (**a**) Manual pruning (reproduced under CC By 4) [47]; (**b**) Manual apple harvesting (reproduced with permission) [67]; (**c**) Manual mango harvesting with a pole (reproduced under CC By 4) [57]; (**d**) Manual rice harvesting [68]; (**e**) Manual rubber harvesting (reproduced under CC By 4) [64].

Agricultural tasks can be divided into several main categories: ground preparation, planting, weeding, pesticide or insecticide spraying, fertilizing, pruning, harvesting, sorting, and forestry operations [36,37,39,40,45,47,60,61], some of which are depicted in Figure 3. All these tasks involve activities that have a high probability of causing WMSDs. Crops are listed in Table 1 with their associated tasks and the probable causes of WMSDs as identified in the literature. When considering the affected areas of the human body, it is evident that the main activities identified in agriculture affect different body parts with varying degrees of severity [43,62,63,64,65]. This is primarily due to the compound and complex motions involved in agricultural activities [66,67]. Therefore, it is vital to propose practical solutions that can be applied to individual tasks.

## 4. Exoskeleton Technology

The concept of modern exoskeletons is closely related to robotics, and thus, they are identified as robotic exoskeletons. Researchers have been involved in this field for a considerable amount of time and have developed new technologies. According to Romero et al. [79], “Exoskeletons are cyber-physical systems with human-machine interfaces which allow humans to build human-automation symbiosis work systems”. However, a simpler explanation of exoskeletons is that they are “super suits or systems that expand or augment a person’s physical abilities” [80]. Wearable exoskeletal implementations mainly focus on reducing the disadvantages associated with manual labor, such as the high risk of WMSDs and physical stress on human workers, enabling safe, efficient, and effective task completion [79]. One of the main objectives in designing an exoskeleton is to reduce the risks of WMSDs by taking off part of the physical stress act on human workers.

Exoskeletons keep the human operator in control [81], making them highly suitable for application in complex agricultural tasks. Exoskeletons can be categorized in various ways [82,83]. Firstly, exoskeletons can be classified based on their activation method. They can be active, requiring a separate power source to actuate, or passive, not requiring a separate power source to actuate. Secondly, exoskeletons can be categorized according to the targeted area of the human body they support [82]. These exoskeletons primarily aim to support major joints or muscles in the human body, such as the shoulder, elbow, upper back, lower back, hips, fingers, wrists, elbows, and knees [82,83]. In addition, exoskeletons can selectively support specific human movements, such as standing, walking, bending, or overhead work [82,84]. Over time, the development of exoskeletons has become industry-specific. Therefore, they can also be categorized based on the intended industry they serve. Military, medical, manufacturing, and eldercare are among the notable industries where modern exoskeletons are focused on assisting [83]. However, in this review, rehabilitation, surgical, and medical-related exoskeletons are excluded as the authors believe that their target task and operational environments do not align with the requirements of agriculture.

When considering active exoskeletons, the majority of them utilize motors as their primary actuators [17,30,85,86,87,88,89,90,91,92,93,94,95,96,97,98]. These motors can provide precision control, enabling better assistive motion capabilities, while hydraulic and pneumatic actuators have also been used in certain heavy-duty applications [30,86,90,91,93]. Active exoskeletons are equipped with additional motion sensors and advanced control systems for real-time controls, and thus they can assist humans with a more “natural sensation”. However, this may lead to dependency on it and overconfidence, increasing the risk of injuries [99,100]. Another significant drawback of active exoskeletons is the operational time. As active exoskeletons depend on a power source, usually a battery, their work duration is limited, which is not desired by agricultural operations. Further, additional challenges have been raised in the maintenance of active skeletons owing to their complex mechanism that may not be favorable in relatively harsh agricultural working conditions [101]. Agricultural work commonly takes place in wet environments, such as the cultivation of rice [40,62], which poses electrical hazards for active exoskeletons. High daytime temperatures of open agricultural land can adversely affect the operation of complex active exoskeletons [40,56]. Moreover, financial burdens arising from expensive electronics in active exoskeletons make them unaffordable for underprivileged or financially strained agricultural workers.

On the other hand, passive exoskeletons have a clear potential to assist agricultural tasks in real-world situations, overcoming the disadvantages of active exoskeletons, such as complexity and operational time [102]. These passive exoskeletons primarily employ elastic fabric materials [9,20,21,23,28,103,104,105,106,107,108,109,110,111] or spring damper systems [20,31,32,103,112] as their main passive assistive element. Hence, their main advantages are simple maintenance and operation without limitations on work duration [18,21,24] or risk of electrocution. When considering the maintenance, it can be identified that most of these passive exoskeletons will only require a change to the passive element. Unlike an active exoskeleton, it will not require any specific knowledge or skill, and thus, it would be much easier for agricultural workers to maintain passive exoskeletons. Fabric-based passive exoskeletons are well suited for agricultural tasks as they allow considerable freedom of movement for the wearer, and they are simple and easy to wear (no rigid components [9,23,105,106,107,108]. However, fine-tuning and precision controls could be compromised due to the use of mechanical systems. Also, passive exoskeletons may not support complex motions or facilitate multiple tasks due to their inherent construction limitations, and a lack of a real-time closed-loop control system. Moreover, a rigid exoskeleton (specifically the ones with spring damper systems) might limit the free motion of the wearing worker and add additional weight to them [99].

Exoskeletons designed for other industries or tasks other than agriculture may require significant modifications and extensive testing before applying them to agricultural applications. As an example, exoskeletons developed for military purposes often tend to be too bulky and impractical for simple agricultural tasks [113]. Furthermore, human power amplifying exoskeletons such as the Human Universal Load Carrier (HULC) [30,93] would not be suitable for agricultural activities as these are complex and bulky. Cutting-edge solutions like augmentative controlled robots with the assistance of exoskeletons [94] could be excessive for relatively simple agricultural tasks. Exoskeletons developed for various industries to support related tasks are listed in Table 2.

## 5. Findings, Challenges, and Considerations

Since the potential causes of WMSDs in agriculture and the various potential exoskeletons have been explored, it is now vital to evaluate the capability of these exoskeletons to assist agricultural workers during the identified labor-intensive tasks. This evaluation can be carried out by considering the specific areas and motions targeted to be supported by the exoskeletons and their impact on the human body during agricultural activities. Most of these exoskeletons were not originally developed for the agricultural industry and are still in the research stage with ongoing efforts to optimize their performance [31,32,89,90,91,92,95,96,105,106,107,109,110,111,112,116,123]. Amongst the identified agricultural tasks, lifting tasks can be directly supported by exoskeletons as the design of several exoskeletons was aimed specifically for this purpose [9,17,20,23,25,30,88,92,94,98,103,105,106,107,108,109,110,111,112,114,122]. It is clear that the best solution for overcoming the risks of WMSDs is to have correct postures [3,4]. As exoskeletons provide a rigid frame, it is possible to use that frame to prevent workers from working in incorrect postures. Repetitive tasks can also take advantage of these exoskeletons as they absorb partial loads from the musculoskeletal system [28,104]. Most importantly, tasks that involve a static posture along with repetitive or random motions should have the most advantages from these investigated exoskeletons. While there may not be clear innovations for tasks involving climbing, reaching, and twisting motions [39,47,52,54,62], an exoskeletal attachment at the end of an agricultural worker’s hand could eliminate the risks associated with reaching and twisting in the wrist.

Several key factors need to be considered to identify suitable exoskeletons for agriculture. Active exoskeletons may not be the best solution, as was discussed in the literature review section. Similarly, hydraulic and pneumatic exoskeletons may be unsuitable as they tend to be bulky and complex for simpler agricultural tasks. Further, active military exoskeletons designed for robustness are not practical for basic agricultural tasks. The exoskeletons at the conceptual level cannot be used until they are fabricated and field-tested properly. Moreover, full-body exoskeletons [40,56,99,100,101] and augmentation-based exo-robots [94] are not suitable for agricultural tasks as they are extremely expensive, impractical, complex, and bulky. After considering the factors discussed above, Table 3 summarizes the potential exoskeletons that can assist in agricultural tasks.

Notably, most of the exoskeletons aimed at supporting upper limbs are able to support agricultural tasks such as hand picking, stick harvesting, pruning, and overhead tasks. It is observed that upper limb exoskeletons are primarily designed to support static postural tasks such as gravity compensation [127]. Agricultural tasks that involve upper limb activities often require repetitive motions. Thus, the applicability of the suggested exoskeletons needs to be assessed for repetitive task assistance. On the other hand, exoskeletons supporting the back, knees, and lower limbs listed in Table 3 can support lifting tasks common in agriculture. When it comes to tasks with back flexion, it could cause a higher degree of postural errors. However, exoskeletons partially absorb tensions in the musculoskeletal system, resulting in a reduced risk of WMSDs [18,21,22,24,31,32,103,114,115,125]. However, the use of exoskeletons to support back flexion requires systematic evaluation as it has not been performed in scientific literature. This is because their assistive method (elastics, spring loads, etc.) might cause more strain on the already exhausted muscles.

Most of the commercially available exoskeletons [9,18,19,21,22,23,24,25,26,27,32,103,105,106,108,114,115,117,120,122,125,128] found in this review can be recommended for agricultural tasks. However, a few of the commercially available exoskeletons lacked detailed information on their operational duration, maintenance, and costs, leading to their exclusion from the final recommendation. The main body parts supported by the filtered exoskeletons in Table 3 are either the back [9,20,23,25,103,105,106,107,108,109,110,114,122,126] or upper limb [18,19,21,22,24,27,29,31,32,103,111,114,115,117,120,125], targeting overhead and lifting tasks [18,19,21,22,24,27,29,31,32,103,111,114,115,117,120,125]. While there are similarities between areas in the human body/specific tasks that exoskeletons are targeted for supporting and areas in the human body/specific tasks in agriculture that are high in potential for causing WMSDs, the complexity of work requirements in agriculture varies significantly. Specifically, overhead-supporting exoskeletons are designed to support the shoulder joint for postures where the hands are held statically at or above shoulder level [18,21,22,103,115,125]. Overhead agricultural tasks often require repetitive shoulder motion [39,47,48,62,63,65,74,129]. Hence, these suggested upper limb-supporting exoskeletons need thorough evaluation before applying in a dynamic agricultural environment. The back supporting exoskeletons, on the other hand, tend to support lifting tasks [9,20,23,25,103,105,106,107,108,109,110,114,122,126], which align with agricultural needs. However, agricultural workers require assistance more on tasks involving bending or leaning forward postures [39,43,52,54,56,62,64,74,75,76]. The suggested, existing exoskeletal solutions have the potential to assist the above-mentioned back flexion tasks. There is still a possibility of having negative impacts because agricultural tasks involve motions such as twisting and holding in various back flexed postures, which exoskeletons are not designed for [7,45,51,53]. Unlike other industries, such as manufacturing and logistics, for which the current exoskeletons are primarily designed, the agricultural field demands support for more dynamic motions in its activities. However, there are a few exoskeletons that can support dynamic movements, such as “Three-layer Fabric Mechanism and Assistive Suit” [98] and some commercial ones [16,18,20,23,24]. Nonetheless, the majority of existing exoskeletons may not satisfy all the support requirements arising from complex and various motions that vary with different crops and regions. Thus, there are ample opportunities for researchers to develop novel solutions that can be of help to different agricultural tasks. It is possible to use existing/commercially available solutions and further develop them to achieve better results [9,21,22,23,25,31,32,94,96,97,98,99,118,119].

**Figure 4:**
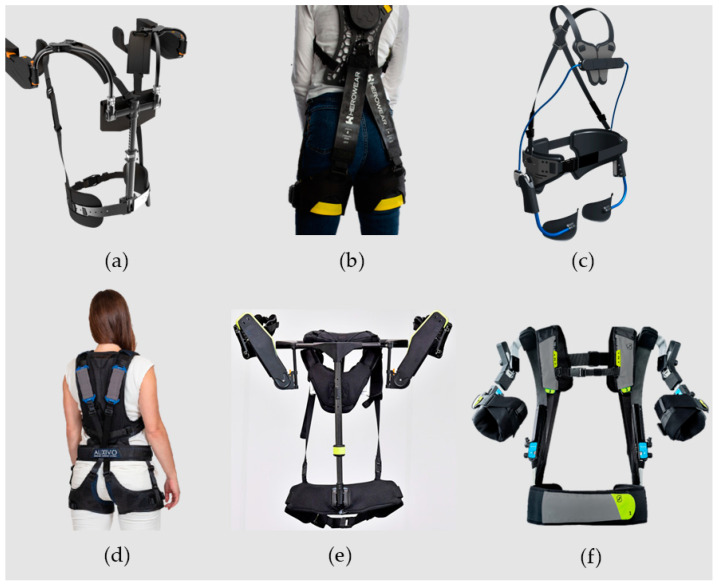
Commercially available exoskeletons (**a**) Levitate Exoskeleton [28,104]; (**b**) Herowear [20,103]; (**c**) Laevo V2.57 [9,23,108]; (**d**) Auxivo Lift Suit [16,105,106]; (**e**) H-Vex [22,115]; (**f**) Skelex 360 [24,114] (pictures courtesy of respective manufacturers).

In consideration of applicability and suitability, agricultural tasks can be assisted by most of the commercially available passive exoskeletons (Figure 4). These passive exoskeletons are mostly lightweight as they do not have active equipment such as electric motors, batteries, hydraulic actuators, or pneumatic actuators. Therefore, the additional weight on a laborer is minimal; thus long duration of fatigueless operation is possible [9,18,19,21,22,23,24,25,26,27,32,103,105,106,108,114,115,117,120,122,125,128].

Additionally, most of the commercially available passive exoskeletons have flexible or semi-flexible, non-rigid structures [9,18,19,21,22,23,24,25,26,27,32,103,105,106,108,114,115,117,120,122,125,128]. Hence, their user can easily wear and use the exoskeleton without limiting the free motion capability, which is vital for working in agricultural fields. Also, all the commercially developed exoskeletons have a comprehensive user manual in which manufacturers have given all the necessary information on how to use, repair, and service the equipment [16,18,19,20,21,23,24,25,26,27,28]. In many cases, manufacturers are willing to provide the necessary training required for the use of the exoskeleton; thus, it will allow the workers to have a proper understanding of the machine, how to use it, and what to do with it.

While several promising exoskeletons are currently under development in the research stage [29,31,32,107,109,110,111,126,130], the lack of information and real-world testing poses a challenge in recommending these to the agricultural industry. Customizing the current exoskeleton may raise another issue related to the affordability of the exoskeletons for agriculture. The modification of already expensive exoskeletons to meet the needs of agricultural activities may add an additional financial burden to economically disadvantaged countries in which numerous WMSDs and associated cases are reported [11,39,43,48,52,54,57,62,64,65,70,71,73,74,77,129].

The review reveals that the majority of agricultural tasks related WMSDs have been reported from South and South East Asian countries [11,39,43,48,52,54,57,62,64,65,70,71,73,74,77,129], whereas exoskeleton research is driven by countries in East Asian, North American, and European regions for non-agricultural tasks [9,18,19,20,22,23,24,25,27,29,31,32,103,108,111,114,115,117,120,122,125,131]. Ironically, developing countries have faced more WMSDs in their agriculture industry, while developed countries are pioneering exoskeleton development. In this case, this mismatch of WMSD perseverance and exoskeleton development seemingly has not assisted the countries and regions that require proper help.

In the majority of the exoskeleton designs and developments, it appears that a crucial aspect has been neglected as researchers have prioritized mimicking human anatomy [132,133]. Given that exoskeletons are wearable, human-in-the-loop devices, it is imperative to integrate human perspectives considering both the mental and physical aspects [10,134,135]. When considering physical aspects, an exoskeleton should not be heavy, bulky, or limit the worker’s mobility and motion. Also, it must be easy to use without worrying about work durations, electric shocks, and other mobility-related hazards such as excessive joint actuation [5,84,113]. Most importantly, exoskeletons should be able to be worn easily and increase the efficiency of the worker. Therefore, it is worth noting that exoskeletons constructed with fabric/elastic materials are more suitable for agricultural tasks, mainly due to their ability to simplify the work process. They are easy to wear and remove, lightweight, and offer a comfortable fit. On the other hand, the psychological effects of using exoskeletons need to be considered. According to surveys found in the literature [99,133], the perception of workers on exoskeletons is not positive. Workers seem to have doubts about the risks involved while using exoskeletons, specifically risks with misalignments, unintended motion, user errors, skin injuries, vibrations, and electrical faults [99]. On the other hand, it can be observed that workers feel threatened or insecure about their jobs because of exoskeletons [133]. Thus, the introduction process of exoskeletal solutions for any workplace would require a very well-planned process.

According to the literature survey on WMSDs, it is evident that there are numerous reports of agriculture-related WMSDs from different regions of the world. A subset of these studies revealed that harvesting tasks are responsible for the majority (51.9%) of the WMSDs [30,94,97]. Also, according to the findings, tasks involving a stooping posture are repetitive in nature and are the main causes of WMSDs, and most of the time, both of those can be observed in the majority of agricultural tasks. In order to enhance the agricultural industry and the wellbeing of workers, it is of utmost importance to implement efficient intervention measures.

When considering the exoskeletons in Table 3 with high potential for assisting agricultural workers, a few key patterns can be recognized. Most of the commercially available upper limb exoskeletons seem to follow a similar design. A rigid frame with a passive actuator is used to translate upper limb loads to hips to provide assistance to the shoulders. All the exoskeletons have focused on providing a maximum range of motion to the shoulder joints, too. Moreover, manufacturers are actively trying to lower the weight of these exoskeletons to minimize user discomfort. Some of the exoskeletons, such as H-Vex [22,115] and ShoulderX by Suitx [27,117], also provide additional neck support, which is an additional advantage when performing overhead tasks. Most upper limb exoskeletons, including those still in the research phase, consider user fit, too. Each of these upper limb exoskeletons offers some degree of customization to ensure a proper fit for the user. A pole-harvesting support exoskeleton [29], the only agricultural task-focused exoskeleton, appears to be relatively heavy as it is still in the early stage of development.

Back support exoskeletons have similar trends to the upper limb exoskeletons. All the commercial exoskeletons adopted a similar design language (Figure 4 b, c, and d). These are designed to be worn over the shoulders at the back and connect to the thighs. According to the manufacturers, these exoskeletons are able to provide a considerable amount of support while performing lifting tasks, making them high potential for similar agricultural tasks. Fabric-based back exoskeletons, such as the three-layer Fabric Mechanism, Assistive Suit [107], Industrial Passive Waist-assistant Exoskeleton IPWE) [126] and carbon fiber composite link-based VT-Lowe’s Exoskeleton [109,110] do not provide technical specifications to compare with commercially available exoskeletons, although they have performed exceptionally well in experiments.

## 6. Regulatory and Ethical Considerations

Due to their direct interaction with human laborers, rigorous regulations and ethical considerations are required for using exoskeletons. Some research is dedicated to exploring ethical, social, legal, and safety factors [132,133,135,136,137], whereby the necessity of regulatory frameworks is identified for this field. Since most exoskeletons are still in the research stage, commercial adoption is not wide-spread. Therefore, the development of legal regulations and ethical considerations is pertinent and timely. These ethical considerations should arise from the initial phase of the exoskeleton design, and consent must be sought from all involved parties before human data can be collected. In addition, it is essential to convey that humans retain control over activities during the use of exoskeletons. Existing standards by the International Organization for Standardization (ISO), such as ISO 13482:2014, should be considered when developing and implementing exoskeleton-based solutions in the real world [127].

Currently, there are no legal and/or standardized regulations for exoskeleton developments or use cases. Thus, researchers must actively seek the perspectives of stakeholders and end-users to ensure usability, safety, and acceptability when developing an exoskeleton, thereby upholding the significance of existing human factors. With the commercial application of exoskeletons in agriculture or any other industry, the workplace dynamics may change, and it is required to continuously re-evaluate working conditions such as work time, rest interval, personal protective equipment, workload distribution, maintenance protocols, and hazard management.

## 7. Future Trends and Supporting Technologies for the Development of Exoskeletons for Agriculture

Developing an exoskeleton is a tedious and long process. Most of the exoskeletons presented in this paper have been through a few iterations to achieve their intended assistive motions and supports. Based on the current development, passive exoskeletons are mostly suited for agricultural tasks. However, this does not imply that active exoskeletons are out of consideration. With the development and advancement of electronics and battery technology, it is possible to develop light power actuators and high-capacity batteries that remove the major hurdle of active exoskeletons, such as limited operational time and their weight.

However, novel low-power, highly efficient, and modular-active actuators are being developed [34,138,139,140,141,142]. Specifically, hydraulic actuators, as described in [138], have a high potential to support static postural tasks seen in agriculture with minimal power consumption. Cluch-based actuation of the spring in a knee exoskeleton, as presented in [140], is another novel invention for exoskeletons. This enables sophisticated support for the user only when needed without the user’s involvement. Soft actuators are another emerging type that can further enhance exoskeletons for agricultural tasks [143]. Soft actuators are generally light and flexible, making exoskeletons equipped with these actuators suitable for complex agricultural movements. Thus, further developments in soft actuators will introduce more variety of exoskeletons in agriculture.

One of the main overlooked areas when considering exoskeletons is the sensors that can be used to either test or control exoskeletons. Although passive exoskeletons, which are ideal for agricultural tasks, are not equipped with built-in control sensors, most passive exoskeletons were developed and tested with Electromyography (EMG) sensors. Accurate EMG signals can reflect the true effects of an exoskeleton on a human body. On the other hand, camera-based motion capture systems play a major role in developing exoskeletons. These systems are able to capture human motion for further analysis. Specifically, these motion capture systems are essential in developing exoskeletons for complex agricultural tasks. Inertial measurement unit (IMU) sensors combined with camera-based motion capture systems provide highly accurate motion data, allowing their user to analyze motions more precisely [34]. Encoders are another important sensor that can be used to control and test exoskeletons. If an active exoskeleton is used for an agricultural task, these sensors will provide precise joint angles so that the actuator can provide the necessary support. Strain and pressure sensors could provide load-related data, making it possible for the exoskeleton to provide necessary supportive forces to the user [34].

## 8. Conclusions and Outlook

WMSDs in agriculture are a global concern demanding immediate attention. To this date, most global food producers still rely heavily on human labor for their agricultural operations. Consequently, the effects of WMSDs hold a potential risk for the global labor force and food production. Despite the emergence of fully autonomous machinery designed to replace human labor, they are still in the preliminary phase of implementation and, thus, are not yet viable for real-world agricultural tasks. Under these circumstances, exoskeletons have been identified as promising candidates for providing a human-centered, easy-to-use, and practical solution for reducing WMSD-related risks in agriculture. Particularly, this study reveals that fabric-based passive exoskeletons are effective and economical in supporting various agricultural activities that involve shoulder motion, bending, stooping, and kneeling. These types of exoskeletons are designed to support major joints and muscles used in agricultural tasks while reducing the risk of WMSDs without interfering with the dynamic motions of agricultural workers. The existing exoskeletons designed for other industries were reviewed in this study to investigate their suitability for agricultural applications. Most current exoskeletons, which are not specifically developed for agricultural tasks, need to be further evaluated, modified, and field-tested prior to wide-spread adoption. Moreover, agricultural countries that are heavily reliant on human labor should focus more on developing their own assistive solutions. Otherwise, these countries may have to use existing exoskeletons from developed countries, which may be unaffordable. Developing their exoskeletal solutions should allow these countries to develop solutions that can be specific to their agricultural tasks and environments, and hence, more effective implementations can be attained.

## Figures and Tables

**Table 1:** Main agricultural tasks and WMSD possibilities.

Agricultural Task	Crop	Probable WMSD Causes	Affected Area of the Body	Reference
Ground Preparation	Rice	Awkward Postures, Heavy Lifting, Repetitive Motion	Neck, Shoulder, Spine	[39,40]
	Potato	Repetitive Motion, Awkward Postures	Back, Upper Limbs	[41]
Planting	Pineapple	Prolonged and Repetitive Awkward Postures	Back, Upper Limbs, Lower Limbs	[42,43]
	Rice	Awkward Posture (bend and work), Repetitive Motion	Knees, Back, Neck, Wrist	[39,62]
	Potato	Awkward Posture (bend and work), Repetitive Motion	Back, Neck, Knees	[41]
Weeding/Pesticide or Insecticide Spraying/Fertilization/Pruning	Pineapple	Awkward Postures	Back, Lower Limbs	[42]
	Rice	Awkward Posture (Manual Weeding),	Upper Limbs (Shoulder), Lower Limbs, Back, Wrist, Neck	[39]
		Repetitive Motion (pesticide and weedicide spraying)	Upper Limbs (Wrist), Back, Lower Limbs	
	Peach	Climbing, Awkward Posture, Repetitive Motion (Pruning)	Back, Upper Limbs (Wrist, Fingers)	[47]
		Repetitive Motion (Pesticide Spraying)	Upper Limbs, Back, and Lower Limbs	
	Grape	Repetitive Motion, Awkward Posture (Pruning)	Upper Limbs (Wrist, Fingers)	[69]
Harvesting	Rice	Awkward Posture (bend and work), Repetitive Motion (trunk twisting, cutting by hands), Heavy Load Lifting (moving the harvest around)	Neck, Trunk, Shoulder, Upper and Lower Back, Knees	[39,54,62]
	Rubber	Awkward Posture (squatting, kneeling), Prolonged Standing, Heavy Lifting, Long Duration of Work	Back, Lower Limbs	[64]
	Mango	Awkward Posture, Repetitive Motion (harvesting with a pole)	Upper Limbs (shoulder), Lower Back, Upper Back	[57]
	Oil Palm	Repetitive Motion, Awkward Posture (harvesting with a pole)	Upper Limbs, Lower Back, Upper Back	[70,71]
	Grape	Prolonged Statical Posture, Repetitive Motion	Upper Limbs (shoulder, wrist, fingers)	[72]
	Apple	Awkward Posture, Repetition	Lumbar, Knee, Shoulder, Neck	[57,73]
	Coffee	Awkward Posture and Loading Conditions, Prolonged Work Duration	Back, Shoulders, Wrist, Fingers	[74]
	Cauliflower, Broccoli, Iceberg, Strawberry	Awkward Posture (bend and work), Repetition	Back, Wrist, Fingers	[75]
	Sugarcane	Repetitive Motion, Heavy and Awkward Lifting	Back, Upper Limbs	[52]
	Lychee-Longan	Climbing, Reaching, Repetitive Motion,	Upper Limbs (wrist, hand, fingers, shoulder), Neck	[76]
	Peach	Climbing, Static Awkward Postures	Upper Limbs	[47]
	Sweet Potatoes	Awkward Posture (bend and work), Prolonged Work Duration, Repetitive Motion	Back, Upper Limbs, Lower Limbs	[56]
	Pineapple	Awkward Postures (bend and work), Heavy Lifting	Lower Back, Knee	[43]
Sorting	Mango	Repetitive Motion	Shoulder, Wrist, Elbow	[57]
	Hazelnut	Repetitive Motion,	Shoulder, Wrist, Elbow	[65]
	Rice	Awkward Posture (bend forward, knees bent, neck bent), Repetitive Motion (upper limbs)	Back, Knees, Upper Limbs	[39]
	Peach	Static Awkward Postures (squatting, kneeling or sitting low)	Knees, Lower Back	[47]
Forestry Operations	N/A	Repetitive Motion, Heavy Lifting, Awkward Postures	Whole body	[76,77,78]

**Table 2:** Summary of exoskeletons found in literature.

Supporting Area of the Body	Specific Area of Support	Name/Made	Power	Industry	Country of Origin	Year	Tasks That Can Be Supported
Upper Body	Upper Limb (Shoulder)	Ekso EVO [20,103].	Passive (Spring-Based Actuator)	Muti-Purpose (Tested on Construction industry)	USA	2023	Overhead Works, Static Shoulder Position Holding Taks.
		Hilti Exo-001 [21,103].	Passive (Straps)	Muti-Purpose (Tested on Construction industry)	USA	2023	Overhead Works, Static Shoulder Position Holding Tasks.
		Armored 3DoF Shoulder Exoskeleton [85]	Active (Motors)	In research stage (Military)	Spain	2020	Shoulder assistance
		Model-based Biomechanical Exoskeleton [112]	Passive (Springs)	In research stage	Germany	2022	Heavy Lifting (Up to shoulder height)
		PULE (Passive Upper Limb Exoskeleton) [32]	Passive (Gas Spring)	In research stage (Agriculture)	China	2021	Arm Lift Task Assistance
		TasKi [31]	Passive (Spring)	In research stage (Agriculture)	Japan	2019	Overhead Work Assistance (Arm lifting and posture holding tasks)
		Skelex 360 [24,114]	Passive (Springs)	Multiple Industries	The Netherlands	2020	Overhead Task Assistance, Static Elevation of Arms Assistance
		Pole harvesting support exoskeleton [29]	Passive (Springs)	In research stage	Malaysia	2021	Upper Limb Motion Assist (in harvesting with a pole)
		H-Vex [22,115]	Passive (Springs)	In research stage	Korea	2019	Overhead Task Assistance
		H-Pulse [116]	Semi Passive (Springs and Active Support Control)	In research stage	Italy	2020	Overhead Task Assistance
		ShoulderX by Suitx [27,117]	Passive (Springs)	Multiple Industries	USA	2020	Overhead Task Assistance
		Levitate exoskeleton [28,104]	Passive (Custom spring- and pulley-based actuator systems)	Multiple Industries	USA	2017	Static Elevation of Arms Assistance, Repetitive Motion Assistance
	Upper Limb (Arm)	Static upper limb activity supporting exoskeleton [118]	Passive (Springs)	In research stage	Switzerland	2018	Upper Limb Static Postures Assistance, Overhead Work Assistance
		Parallelogram type Exoskeleton [119]	Passive (Spring)	In research stage	Switzerland	2016	Overhead Work Assistance, Hand Raising Task Assistance (Gravity compensation)
	Upper Limbs (Shoulder and Elbow)	Hapo MS [19,120]	Passive (Springs)	Multiple Industries	France	2023	Overhead Task Assistance
	Upper Limb (Elbow)	Power-Assist Exoskeleton [86]	Active (Pneumatic)	In research stage	China	2014	Power Assistance
	Upper Limb	No name, design and lab testing only [87]	Active (Motos and Gears)	In research stage	Japan	2018	Lifting, Posture Support
	Fingers	Double-Acting Soft Actuator (DASA)-Based Robotic Glove [121]	Active (Pneumatic)	In research stage	China/Honk Kong	2023	Finger Extension/Flexion
Back	Back (Lower and Upper	Hero Wear Apex [20,103].	Passive (Elastic Straps)	Multipurpose (Tested on Construction industry)	USA	2023	Lifting Tasks, Forward Bending Tasks, Push and Pull Taks.
	Lower Back	LiftSuit v2.0 (Auxivo AG) [16,105,106]	Passive (Textile Springs)	In research stage	Switzerland	2022	Heavy Lifting, Forward Leaning Posture Assistance
		Three-layer Fabric Mechanism, Assistive Suit [107]	Passive (Elastic Fabrics)	In research stage	Japan	2009	Posture Support
		IPWE (Industrial Passive Waist-assistant Exoskeleton) [107]	Passive (Elastic Straps)	In research stage	China	2020	Lifting Assistance
		Laevo 2.0 [9,23,108]	Passive (Elastic Fabrics)	Multipurpose	The Netherlands	2019	Lifting Assistance
		VT-Lowe’s Exoskeleton [109,110]	Passive (Carbon Fibre Legs)	In research stage	USA	2019	Lifting Assistance
		BACKX by Suitx [25,114,122]	Passive (gas springs)	Multipurpose	USA	2020	Lifting Assistance
		Dynamic Lifting aid Exoskeleton [88]	Active (Motors)	In research stage	Europe (Ireland, The Netherlands, Italy)	2017	Lifting Assistance
	Back and Upper Limb	Ez-UP [111]	Passive (Deformable and Non-Deformable Belts with Quadrilateral structured Elastic Fabric)	In research stage (Lab Testing)	Japan	2020	Lifting, Forward Bent Work Posture Support
Lower Body	Lower Limbs	exoskeleton for static conditions using Indian anthropometric considerations [123]	Passive (Metal links)	In research stage	India	2020	Static Standing Assistance
		MIT lower-body exoskeleton [30]	Active (Motor)	Military	USA	2009	Heavy Lifting, Load Carrying
		Lower Limb Exoskeleton [89]	Active (Motors)	In research stage	Japan	2019	Walking Assistance
		Lowerlimb energy harvesting and transmission exoskeleton (EHTE) [124]	Passive (Flat Spiral Springs)	In research stage	China	2021	Walking Assistance
		AWGAS (Assistive Wearable Gait Augment Suit) [90]	Active Passive (Pneumatic and Gel Muscles)	In research stage	Japan	2018	Gait/Walking Assistance, Postural Assistance, Bent (knee) task assistance
	Knees	Endoskeleton Type Knee Joint Assist [91]	Active (Pneumatic)	In research stage	Japan	2021	Posture Support (half sitting and crouching)
		Knee exoskeleton [92]	Active (Motors)	In research stage	Japan	2016	Lifting from Crouch Position
	Lower Limbs/Back	HULC [30,93]	Active (Hydraulic)	Military	USA	2009	Heavy Lifting, Load Carrying (Enhance load capacity)
		CRAY X [17,94]	Active (Motors)	Manufacturing	Germany	2019	Lifting heavy loads
		Model A/Model Y [94]	Active (Motors)	Various Industries that handle goods	Japan	2019	Heavy Lifting, Posture Support
	Lower Back/Top of Lower Limbs	No name, Design only [95]	Active (Motors)	In research stage	India	2022	Heavy Lifting
	Hip, Knee	Non-Exoskeletal Structure [96]	Active (Motors)	In research stage	Japan	2014	Walking Assistance, Power Assistance
Whole body	-	Raytheon/Sarcos exoskeleton [30]	Active (Motors)	Military	USA	2009	Heavy Lifting
	Separate modules for different areas	HAL [30,94,97]	Active (Motors)	Multipurpose	Japan	2019	Lifting, Posture Support
	-	Tokyo University of Agriculture and Technology—Exoskeleton [30]	Active (Motors)	Agriculture (Support for elderly workers)	Japan	2009	Posture Support
	-	Guardian XO and Guardian XO MAX [94,98]	Active (Motors)	Manufacturing	USA	2019	Heavy Lifting
	Augmented	Guardian GT [94]	Active (Motors)	Manufacturing	USA	2019	Multiple tasks and can be remotely operated.

**Table 3:** Exoskeletons that have the potential to assist in agricultural tasks.

Area of the Human Body (Affected/Supported)	Availability	Exoskeleton	Features/Specifications	Possible Agricultural Activities
Upper Limb (Shoulder)	Commercially Available	Ekso EVO [18,103,125]	○ Exoskeleton weight—4.3 kg ○ Adjustable support (2.25 kg–6.8 kg) ○ Allows full range of motion (shoulder and back)	○ Grape, Apple, Coffee, Peach Harvesting (by hand) ○ Mango/Oil Palm Harvesting (by stick) ○ Grape Pruning ○ Forestry operations that involve overhead tasks ○ Sorting tasks that involve static shoulder posture (Hazelnut, Mango)
		Hilti Exo-001 [21,103]	○ Exoskeleton weight—under 2 kg. ○ Allows full range of motion (shoulder) ○ Best suited for overhead tasks.	
		H-Vex [22,115]	○ Exoskeleton weight—2.5 kg ○ Additional neck support ○ Allows full range of motion (shoulder)	
		Skelex 360 [24,114]	○ Exoskeleton weight—2.3 kg ○ Adjustable support (0.5 kg–4 kg) ○ Allows full range of motion (shoulder) ○ One-size-fits-all build	
		ShoulderX by Suitx [27,117]	○ Exoskeleton weight—2.22 kg ○ Allows full range of motion (shoulder) ○ Adjustable to fit different-sized people	
	In Research Stage	PULE (Passive Upper Limb Exoskeleton) [32]	○ Exoskeleton weight—1.9 kg ○ Allows full range of motion	
		TasKi [31]	○ Provides pre-defined assistance for overhead tasks for different individuals. ○ Consists of pre-use procedure to fine-tune the support and exoskeleton	
Upper Limb (Shoulder and Elbow)	Commercially Available	Hapo MS [19,120]	○ Exoskeleton weight—1.3 kg ○ Adjustable support (3 kg or 2 kg) ○ Range of motion with assistance—up to 135in height and up to 180in horizontal ○ One size fit all	
Upper Limb	In the Research Stage but developed specifically for harvesting tasks with a pole/stick.	Pole harvesting support exoskeleton [29]	○ Exoskeleton weight—3.8 kg ○ Supports shoulder flexion in the sagittal plane between 45and 135°. ○ Custom built for harvesting with a pole.	○ Oil palm/Mango harvesting.
Upper Limb/Back	In Research Stage	Ez-UP [111]	○ Exoskeleton weight—0.75 kg. ○ Provide support for the back and upper limbs simultaneously. ○ Fabric-based. ○ Easy to wear.	○ Can support tasks that involve bending and lifting loads. ○ Rice manual planting, weeding, harvesting and sorting. ○ Pineapple planting and harvesting. ○ Potato planting. ○ Sweet potato harvesting. ○ Rubber harvesting. ○ Cauliflower harvesting. ○ Broccoli harvesting. ○ Iceberg harvesting. ○ Strawberry harvesting. ○ Sugarcane harvesting. ○ Peach sorting. ○ Forestry work that requires bending and lifting.
Back (Lower and Upper)	Commercially Available	Hero Wear Apex [20,103]	○ Exoskeleton weight—1.5 kg ○ Provide support at the back over 23 kg. ○ Comes in over 50 different fit combinations. ○ Textile-based and breathable. ○ Can manually activate and deactivate support	
	Commercially Available	Laevo 2.0 [9,23,108]	○ Exoskeleton weight—3 kg ○ Supports hip joint providing 30 Nm max torque at 40bending. ○ Additionally, improves the posture while bending ○ Can manually activate and deactivate support	
	Commercially Available	BACKX (IX Back) by Suitx [25,114,122]	○ 25 kg maximum support in lumbar area. ○ One size fits all. ○ Support ergonomically correct postures.	
	Commercially Available	LiftSuit v2.0 (Auxivo AG) [16,105,106]	○ Exoskeleton weight—0.9 kg ○ Exoskeleton is made out of textile and artificial muscles ○ 21% reduction in peak muscle activity while lifting 6 kg.	
	In Research Stage	Three-layer Fabric Mechanism, Assistive Suit [107]	-	
	In Research Stage	IPWE (Industrial Passive Waist-assistant Exoskeleton) [126]	-	
	In Research Stage	VT-Lowe’s Exoskeleton [109,110]	-	

## Data Availability

No new data were created or analyzed in this study. Data sharing is not applicable to this article.

## References

[1] Marcum J., Adams D. (2017). Work-related musculoskeletal disorder surveillance using the Washington state workers’ compensation system: Recent declines and patterns by industry, 1999–2013, (in eng). Am. J. Ind. Med..

[2] Oakman J., Clune S., Stuckey R. (2019). Musculoskeletal Disorders in Australia. The Latest Research on Work-Related Musculoskeletal Disorders.

[3] Stack T., Ostrom L.T., Wilhelmsen C.A. (2016). Occupational Ergonomics: A Practical Approach.

[4] Violante F., Kilbom A., Armstrong T.J. (2000). Occupational Ergonomics: Work Related Musculoskeletal Disorders of the Upper Limb and Back.

[5] Theurel J., Desbrosses K. (2019). Occupational Exoskeletons: Overview of Their Benefits and Limitations in Preventing Work-Related Musculoskeletal Disorders. IISE Trans. Occup. Ergon. Hum. Factors.

[6] Zhang B., Chen X., Zhang H., Shen C., Fu W. (2022). Design and Performance Test of a Jujube Pruning Manipulator. Agriculture.

[7] Varghese A., Panicker V.V. (2022). Impact of musculoskeletal disorders on various agricultural operations: A systematic review. Sadhana.

[8] Satyajit U., Roberto F., Kim N., Divya S. (2019). The Potential for Exoskeletons to Improve Health and Safety in Agriculture—Perspectives from Service Providers. IISE Trans. Occup. Ergon. Hum. Factors.

[9] Ornwipa T., Stephan M., Divya S., Catherine T. (2020). Potential exoskeleton uses for reducing low back muscular activity during farm tasks. Am. J. Ind. Med..

[10] Omoniyi A., Trask C., Milosavljevic S., Thamsuwan O. (2020). Farmers’ perceptions of exoskeleton use on farms: Finding the right tool for the work(er). Int. J. Ind. Ergon..

[11] Kumaraveloo K.S., Lunner Kolstrup C. (2018). Agriculture and musculoskeletal disorders in low- and middle-income countries. J. Agromedicine.

[12] Karsh B.T. (2006). Theories of work-related musculoskeletal disorders: Implications for ergonomic interventions. Theor. Issues Ergon. Sci..

[13] Benos L., Tsaopoulos D., Bochtis D. (2020). A Review on Ergonomics in Agriculture. Part I: Manual Operations. Appl. Sci..

[14] Barneo-Alcántara M., Díaz-Pérez M., Gómez-Galán M., Carreño-Ortega Á., Callejón-Ferre Á.-J. (2021). Musculoskeletal Disorders in Agriculture: A Review from Web of Science Core Collection. Agronomy.

[15] Nicholas Y. (1890). Apparatus for Facilitating Walking. U.S. Patent.

[16] AUXIVO LiftSuit 2: Support, Anytime, Anywhere. https://www.auxivo.com/liftsuit.

[17] Bionic G. Cray X. https://germanbionic.com/en/solutions/exoskeletons/crayx/.

[18] Eksobionics eksoEVO. https://eksobionics.com/ekso-evo/.

[19] Ergosanté HAPO Front (Formerly MS). https://ergosante.fr/en/exosquelette-leger-hapo-ms/.

[20] HEROWEAR HeroWear Apex Science Overview. https://herowearexo.com/the-science-studies-behind-the-apex-back-exosuit/.

[21] Hilti EXO-S SHOULDER EXOSKELETON. https://www.hilti.com/c/CLS_EXOSKELETON_HUMAN_AUGMENTATION/CLS_UPPERBODY_EXOSKELETON/CLS_SUB_UPPERBODY_EXOSKELETON/r14012433.

[22] Hyundai Hyundai Develops Wearable Vest Exoskeleton for Overhead Work. https://www.hyundai.news/eu/articles/press-releases/hyundai-develops-wearable-vest-exoskeleton-for-overhead-work.html.

[23] Laevo Laevo V2. https://www.laevo-exoskeletons.com/laevo-v2.

[24] Skelex Skelex 360-XFR. https://www.skelex.com/skelex-360-xfr/.

[25] Suitx Suitx Exoskeletons. https://www.suitx.com/backX.

[26] Suitx LegX by Suitx. https://www.suitx.com/legx.

[27] Suitx ShoulderX by Suitx. https://www.suitx.com/shoulderx.

[28] Technologies L. The AIRFRAME. https://www.levitatetech.com/airframe-flex/.

[29] Harith H.H., Mohd M.F., Sowat S.N. (2021). A preliminary investigation on upper limb exoskeleton assistance for simulated agricultural tasks. Appl. Ergon..

[30] Bogue R. (2009). Exoskeletons and robotic prosthetics: A review of recent developments. Ind. Robot..

[31] Yamada Y., Arakawa H., Watanabe T., Fukuyama S., Nishihama R., Kikutani I., Nakamura T. (2020). TasKi: Overhead Work Assistance Device with Passive Gravity Compensation Mechanism. J. Robot. Mechatron..

[32] Wang H.-M., Le D.K.L., Lin W.-C. (2021). Evaluation of a Passive Upper-Limb Exoskeleton Applied to Assist Farming Activities in Fruit Orchards. Appl. Sci..

[33] Takahashi K., Muraoka R., Otsuka K. (2020). Technology adoption, impact, and extension in developing countries’ agriculture: A review of the recent literature. Agric. Econ..

[34] Tiboni M., Borboni A., Vérité F., Bregoli C., Amici C. (2022). Sensors and Actuation Technologies in Exoskeletons: A Review. Sensors.

[35] CCOHS Work-Related Musculoskeletal Disorders (WMSDs). https://www.ccohs.ca/oshanswers/diseases/rmirsi.html.

[36] Osborne A., Blake C., Fullen B.M., Meredith D., Phelan J., McNamara J., Cunningham C. (2012). Prevalence of musculoskeletal disorders among farmers: A systematic review. Am. J. Ind. Med..

[37] Walker-Bone K., Palmer K.T. (2002). Musculoskeletal disorders in farmers and farm workers. Occup. Med. (Oxf.).

[38] Bispo L.G.M., Moreno C.F., Silva G.H.d.O., Albuquerque N.L.B.d., Silva J.M.N.d. (2022). Risk factors for work-related musculoskeletal disorders: A study in the inner regions of Alagoas and Bahia. Saf. Sci..

[39] Mishra D., Satapathy S. (2019). Ergonomic risk assessment of farmers in Odisha (India). Int. J. Syst. Assur. Eng. Manag..

[40] Das B. (2015). Gender differences in prevalence of musculoskeletal disorders among the rice farmers of West Bengal, India. Work.

[41] Das B., Gangopadhyay S. (2015). Prevalence of Musculoskeletal Disorders and Physiological Stress Among Adult, Male Potato Cultivators of West Bengal, India. Asia-Pac. J. Public Health.

[42] Salleh N.F.M., Sukadarin E.H., Khamis N.K., Ramli R. (2019). Pattern of muscle contraction in different postures among Malaysia pineapple plantation workers. IOP Conf. Series Mater. Sci. Eng..

[43] Mohamad Salleh N.F., Hani Sukadarin E. (2018). Defining Human Factor and Ergonomic and its related issues in Malaysia Pineapple Plantations. MATEC Web Conf..

[44] Oakman J., Chan S. (2015). Risk management: Where should we target strategies to reduce work-related musculoskeletal disorders?. Saf. Sci..

[45] Gangopadhyay S., Dev S. (2014). Design and evaluation of ergonomic interventions for the prevention of musculoskeletal disorders in India. Ann. Occup. Environ. Med..

[46] Kee D. (2022). Systematic Comparison of OWAS, RULA, and REBA Based on a Literature Review. Int. J. Environ. Res. Public Health.

[47] Kee D. (2022). Participatory Ergonomic Interventions for Improving Agricultural Work Environment: A Case Study in a Farming Organization of Korea. Appl. Sci..

[48] Akbar K.A., Try P., Viwattanakulvanid P., Kallawicha K. (2023). Work-Related Musculoskeletal Disorders Among Farmers in the Southeast Asia Region: A Systematic Review. Saf. Health Work..

[49] Das B., Gangopadhyay S. (2018). Occupational agricultural injuries among the preadolescent workers of West Bengal, India. Int. J. Adolesc. Med. Health.

[50] Bruno R.D.C., Edgar Ramos V. (2010). Risk factors for work-related musculoskeletal disorders: A systematic review of recent longitudinal studies. Am. J. Ind. Med..

[51] James M.M., John A.M., Diana G.T., Julia F., Ira J., Ed W., Rhonda S., Linda G. (2002). Priority risk factors for back injury in agricultural field work: Vineyard ergonomics. J. Agromed..

[52] Teerasak P., Kessarawan N., Dariwan S., Wongsa L. (2014). Work-Related Musculoskeletal Disorders Among Sugarcane Farmers in North-Eastern Thailand. Asia Pac. J. Public Health.

[53] Osborne A., Blake C., Meredith D., Kinsella A., Phelan J., McNamara J., Cunningham C. (2013). Work-related musculoskeletal disorders among Irish farm operators. Am. J. Ind. Med..

[54] Sa’diyah N., Maksum M., Mulyati G.T. (2021). Reducing MSDs and physical workload of manual-harvesting peasan. The International Conference on Smart and Innovative Agriculture.

[55] Kee D., Haslam R. (2019). Prevalence of work-related musculoskeletal disorders in agriculture workers in Korea and preventative interventions. Work.

[56] Kearney G.D., Allen D.L., Balanay J.A.G., Barry P. (2016). A Descriptive Study of Body Pain and Work-Related Musculoskeletal Disorders Among Latino Farmworkers Working on Sweet Potato Farms in Eastern North Carolina. J. Agromed..

[57] Boriboonsuksri P., Taptagaporn S., Kaewdok T. (2022). Ergonomic Task Analysis for Prioritization of Work-Related Musculoskeletal Disorders among Mango-Harvesting Farmers. Safety.

[58] Momeni Z., Choobineh A., Razeghi M., Ghaem H., Azadian F., Daneshmandi H. (2020). Work-related Musculoskeletal Symptoms among Agricultural Workers: A Cross-sectional Study in Iran. J. Agromed..

[59] Mohamad Rashid Mohamad R., Mohd Amran Mohd D., Mohamad Ikbar Abdul W., Khairanum S., Qarna M., Shazia P. (2022). The Evolution of Ergonomics Risk Assessment Method to Prevent Work-Related Musculoskeletal Disorders (WMSDS). Int. J. Online Biomed. Eng. (Ijoe).

[60] Gangopadhyay S., Das B., Das T., Ghoshal G., Ghosh T., Ara T., Dev S. (2009). Ergonomics study on Musculoskeletal Disorders among female agricultural workers of West Bengal, India. Ergon. SA.

[61] Kee D. (2023). Characteristics of Work-Related Musculoskeletal Disorders in Korea. Int. J. Environ. Res. Public Health.

[62] Vimal V., Kamble R., Pandit S. (2023). Comparative ergonomic assessment of manual harvesting of un-lodged and lodged paddy crops post-tropical cyclone in India. Int. Arch. Occup. Environ. Health.

[63] Chokprasit P., Yimthiang S., Veerasakul S. (2022). Predictors of Low Back Pain Risk among Rubber Harvesters. Int. J. Environ. Res. Public Health.

[64] Colantoni A., Cecchini M., Monarca D., Bedini R., Riccioni S. (2013). The risk of musculoskeletal disorders due to repetitive movements of upper limbs for workers employed in hazelnut sorting. J. Agric. Eng..

[65] Chan Y.S., Teo Y.X., Gouwanda D., Nurzaman S.G., Gopalai A.A. (2023). Simulation of passive exotendon assistive device for agricultural harvesting task. Phys. Eng. Sci. Med..

[66] Anagnostis A., Benos L., Tsaopoulos D., Tagarakis A., Tsolakis N., Bochtis D. (2021). Human Activity Recognition through Recurrent Neural Networks for Human–Robot Interaction in Agriculture. Appl. Sci..

[67] Houshyar E., Kim I.-J. (2018). Understanding musculoskeletal disorders among Iranian apple harvesting laborers: Ergonomic and stop watch time studies. Int. J. Ind. Ergon..

[68] Castelein R.B., Broeze J., Kok M.G., Axmann H.B., Guo X., Soethoudt J.M. (2022). Mechanization in rice farming reduces greenhouse gas emissions, food losses, and constitutes a positive business case for smallholder farmers—Results from a controlled experiment in Nigeria. Clean. Eng. Technol..

[69] Roquelaure Y., Gabignon Y., Gillant J.C., Delalieux P., Ferrari C., Méa M., Fanello S., Penneau-Fontbonne D. (2001). Transient hand paresthesias in Champagne vineyard workers. Am. J. Ind. Med..

[70] Sukadarin E.H., Md Deros B., Mokhtar M.M. (2013). Evaluation of Musculoskeletal Disorders Prevalence during Oil Palm Fresh Fruit Bunches Harvesting Using RULA. Adv. Eng. Forum.

[71] Mohamaddan S., Rahman M.A., Andrew_Munot M., Tanjong S.J., Deros B.M., Md Dawal S.Z., Case K. (2021). Investigation of oil palm harvesting tools design and technique on work-related musculoskeletal disorders of the upper body. Int. J. Ind. Ergon..

[72] Wakula J., Landau K. (2000). Ergonomic analysis of grapevine pruning and wine harvesting to define work and hand tools design requirements. Proc. Hum. Factors Ergon. Soc. Annu. Meet..

[73] Thamsuwan O., Galvin K., Tchong-French M., Kim J.H., Johnson P.W. (2019). A feasibility study comparing objective and subjective field-based physical exposure measurements during apple harvesting with ladders and mobile platforms. J. Agromed..

[74] Silverstein B.A., Bao S.S., Russell S., Stewart K. (2012). Water and Coffee: A Systems Approach to Improving Coffee Harvesting Work in Nicaragua. Hum. Factors.

[75] Pinzke S., Lavesson L. (2018). Ergonomic conditions in manual harvesting in Swedish outdoor cultivation. Ann. Agric. Environ. Med..

[76] Ong-Artborirak P., Kantow S., Seangpraw K., Tonchoy P., Auttama N., Choowanthanapakorn M., Boonyathee S. (2022). Ergonomic Risk Factors for Musculoskeletal Disorders among Ethnic Lychee-Longan Harvesting Workers in Northern Thailand. Healthcare.

[77] Gallo R., Mazzetto F. (2013). Ergonomic analysis for the assessment of the risk of work-related musculoskeletal disorder in forestry operations. J. Agric. Eng..

[78] Phairah K., Brink M., Chirwa P., Todd A. (2016). Operator work-related musculoskeletal disorders during forwarding operations in South Africa: An ergonomic assessment. South. For..

[79] Schettino S., Minette L.J., Andrade Lima R.C., Pedroso Nascimento G.S., Caçador S.S., Leme Vieira M.P. (2021). Forest harvesting in rural properties: Risks and worsening to the worker’s health under the ergonomics approach. Int. J. Ind. Ergon..

[80] Romero D., Stahre J., Wuest T., Noran O., Bernus P., Fasth F.-B.Å., Gorecky D. Towards an Operator 4.0 Typology: A Human-Centric Perspective on the Fourth Industrial Revolution Technologies. Proceedings of the International Conference on Computers & Industrial Engineering (CIE46).

[81] Yang C.-J., Zhang J.-F., Chen Y., Dong Y.-M., Zhang Y. (2008). A Review of exoskeleton-type systems and their key technologies. Proc. Inst. Mech. Eng. Part C J. Mech. Eng. Sci..

[82] Zhang J., Fiers P., Witte K.A., Jackson R.W., Poggensee K.L., Atkeson C.G., Collins S.H. (2017). Human-in-the-loop optimization of exoskeleton assistance during walking. Science.

[83] Gopura R.A.R.C., Kiguchi K. Mechanical designs of active upper-limb exoskeleton robots: State-of-the-art and design difficulties. Proceedings of the 2009 IEEE International Conference on Rehabilitation Robotics.

[84] Fox S., Aranko O., Heilala J., Vahala P. (2020). Exoskeletons. J. Manuf. Technol. Manag..

[85] Danko A.-D., Straka M. (2022). Exoskeletons—Robotic suits improving work in logistics. Acta Logist..

[86] López-Méndez S., Martínez-Tejada H.V., Valencia-García M.F. (2020). Development of an armored upper limb exoskeleton. Rev. Fac. De Ing..

[87] Tang Z., Zhang K., Sun S., Gao Z., Zhang L., Yang Z. (2014). An upper-limb power-assist exoskeleton using proportional myoelectric control. Sensors.

[88] Inoue H., Noritsugu T. (2018). Development of Upper-Limb Power Assist Machine Using Linkage Mechanism—Drive Mechanism and its Applications. J. Robot. Mechatron..

[89] Huysamen K., de Looze M., Bosch T., Ortiz J., Toxiri S., O’Sullivan L.W. (2018). Assessment of an active industrial exoskeleton to aid dynamic lifting and lowering manual handling tasks. Appl. Ergon..

[90] Nomura S., Takahashi Y., Sahashi K., Murai S., Kawai M., Taniai Y., Naniwa T. (2019). Power Assist Control Based on Human Motion Estimation Using Motion Sensors for Powered Exoskeleton without Binding Legs. Appl. Sci..

[91] Thakur C., Ogawa K., Kurita Y. (2018). Active Passive Nature of Assistive Wearable Gait Augment Suit for Enhanced Mobility. J. Robot. Mechatron..

[92] Uchiyama K., Ito T., Tomori H. (2022). Development of Endoskeleton Type Knee Joint Assist Orthosis Using McKibben Type Artificial Muscle. J. Robot. Mechatron..

[93] Naruoka Y., Hiramitsu N., Mitsuya Y. (2016). A Study of Power-Assist Technology to Reduce Body Burden During Loading and Unloading Operations by Support of Knee Joint Motion. J. Robot. Mechatron..

[94] Lockheed Martin B.B. Human Universal Load Carrier (HULC). https://bleex.me.berkeley.edu/project/hulc/.

[95] Bogue R. (2018). Exoskeletons—A review of industrial applications. Ind. Robot.

[96] Chittar O.A., Barve S.B. (2022). Waist-Supportive Exoskeleton: Systems and Materials. Mater. Today Proc..

[97] Tanaka H., Hashimoto M. (2014). Development of a Non-Exoskeletal Structure for a Robotic Suit. Int. J. Autom. Technol..

[98] Cyberdyne What is HAL. https://www.cyberdyne.jp/english/products/HAL/index.html.

[99] SARCOS Guardian XO Full-Body Power Exoskeleton. https://www.sarcos.com/products/guardian-xo-powered-exoskeleton/.

[100] Massardi S., Pinto-Fernandez D., Babič J., Dežman M., Trošt A., Grosu V., Lefeber D., Rodriguez C., Bessler J., Schaake L. (2023). Relevance of hazards in exoskeleton applications: A survey-based enquiry. J. Neuroeng. Rehabil..

[101] Kranenborg S.E., Greve C., Reneman M.F., Roossien C.C. (2023). Side-effects and adverse events of a shoulder- and back-support exoskeleton in workers: A systematic review. Appl. Ergon..

[102] Anam K., Al-Jumaily A.A. (2012). Active Exoskeleton Control Systems: State of the Art. Procedia Eng..

[103] Halim I., Saptari A., Abdullah Z., Perumal P., Zainal Abidin M.Z., Muhammad M.N., Abdullah S. (2022). Critical Factors Influencing User Experience on Passive Exoskeleton Application: A Review. Int. J. Integr. Eng..

[104] Bennett S.T., Han W., Mahmud D., Adamczyk P.G., Dai F., Wehner M., Veeramani D., Zhu Z. (2023). Usability and Biomechanical Testing of Passive Exoskeletons for Construction Workers: A Field-Based Pilot Study. Buildings.

[105] Spada S., Ghibaudo L., Gilotta S., Gastaldi L., Cavatorta M.P. (2017). Investigation into the Applicability of a Passive Upper-limb Exoskeleton in Automotive Industry. Procedia Manuf..

[106] van Sluijs R.M., Rodriguez-Cianca D., Sanz-Morère C.B., Massardi S., Bartenbach V., Torricelli D. (2023). A method to quantify the reduction of back and hip muscle fatigue of lift-support exoskeletons. Wearable Technol..

[107] van Sluijs R.M., Wehrli M., Brunner A., Lambercy O. (2023). Evaluation of the physiological benefits of a passive back-support exoskeleton during lifting and working in forward leaning postures. J. Biomech..

[108] Wan C.L., Ishioka T., Kanda C., Osawa K., Kodama K., Tanaka E. (2022). Development of a Three-Layer Fabric Mechanism for a Passive-Type Assistive Suit. J. Robot. Mechatron..

[109] Koopman A.S., Kingma I., Faber G.S., De Looze M.P., Van Dieën J.H. (2019). Effects of a passive exoskeleton on the mechanical loading of the low back in static holding tasks. J. Biomech..

[110] Mohammad Mehdi A., Jack G., Athulya A.S., Chang S.E., Alan T.A. (2019). A passive exoskeleton reduces peak and mean EMG during symmetric and asymmetric lifting. J. Electromyogr. Kinesiol..

[111] Tech V. Lowe’s and Virginia Tech Develop Exosuit Designed to Help Retail Employees. https://news.vt.edu/articles/2017/05/eng-lowesexosuit.html.

[112] Liao Y.-T., Ishioka T., Mishima K., Kanda C., Kodama K., Tanaka E. (2020). Development and Evaluation of a Close-Fitting Assistive Suit for Back and Arm Muscle–e.z.UP. J. Robot. Mechatron..

[113] Schiebl J., Tröster M., Idoudi W., Gneiting E., Spies L., Maufroy C., Schneider U., Bauernhansl T. (2022). Model-Based Biomechanical Exoskeleton Concept Optimization for a Representative Lifting Task in Logistics. Int. J. Environ. Res. Public Health.

[114] Proud J.K., Lai D.T.H., Mudie K.L., Carstairs G.L., Billing D.C., Garofolini A., Begg R.K. (2022). Exoskeleton Application to Military Manual Handling Tasks. Hum. Factors.

[115] Gull M.A., Bai S., Bak T. (2020). A Review on Design of Upper Limb Exoskeletons. Robotics.

[116] Dong Jin H., KiHyeon B., KyuJung K., Seungkyu N., Dong-hyun L. (2019). A light-weight passive upper arm assistive exoskeleton based on multi-linkage spring-energy dissipation mechanism for overhead tasks. Robot. Auton. Syst..

[117] Grazi L., Trigili E., Proface G., Giovacchini F., Crea S., Vitiello N. (2020). Design and Experimental Evaluation of a Semi-Passive Upper-Limb Exoskeleton for Workers With Motorized Tuning of Assistance. IEEE Trans. Neural Syst. Rehabil. Eng..

[118] Van Engelhoven L., Poon N., Kazerooni H., Rempel D., Barr A., Harris-Adamson C. (2019). Experimental Evaluation of a Shoulder-Support Exoskeleton for Overhead Work: Influences of Peak Torque Amplitude, Task, and Tool Mass. IISE Trans. Occup. Ergon. Hum. Factors.

[119] Huysamen K., Bosch T., de Looze M., Stadler K.S., Graf E., O’Sullivan L.W. (2018). Evaluation of a passive exoskeleton for static upper limb activities. Appl. Ergon..

[120] Ruprecht A., Daniel S., Konrad S.S. (2016). Design of a passive, iso-elastic upper limb exoskeleton for gravity compensation. ROBOMECH J..

[121] Arnoux B., Farr A., Boccara V., Vignais N. (2023). Evaluation of a Passive Upper Limb Exoskeleton in Healthcare Workers during a Surgical Instrument Cleaning Task. Int. J. Environ. Res. Public Health.

[122] Liu H., Wu C., Lin S., Chen Y., Hu Y., Xu T., Yuan W., Li Y. (2023). Finger Flexion and Extension Driven by a Single Motor in Robotic Glove Design. Adv. Intell. Syst..

[123] Kazerooni H., Tung W., Pillai M. (2019). Evaluation of Trunk-Supporting Exoskeleton. Proc. Hum. Factors Ergon. Soc. Annu. Meet..

[124] Abhilash C.R., Sriraksha M., Haq M.A., Narahari N.S. (2022). Design and evaluation of exoskeleton for static conditions using Indian anthropometric considerations. J. Eng. Des. Technol..

[125] Zhou X., Liu G., Han B., Wu L., Li H. (2021). Design of a Human Lower Limbs Exoskeleton for Biomechanical Energy Harvesting and Assist Walking. Energy Technol..

[126] Kim S., Nussbaum M.A., Smets M., Ranganathan S. (2021). Effects of an arm-support exoskeleton on perceived work intensity and musculoskeletal discomfort: An 18-month field study in automotive assembly. Am. J. Ind. Med..

[127] International Organization for Standardization ISO 13482:2014. https://www.iso.org/standard/53820.html#:~:text=ISO%2013482%3A2014%20specifies%20requirements,person%20carrier%20robot.

[128] Zeng D., Qu S., Ma T., Yin P., Gao H., Zhao N., Xia Y. (2021). The Assist Performance Test of Industrial Passive Waist-assistant Exoskeleton on Fatigue during a Repetitive Lifting Task. J. Phys. Conf. Ser..

[129] Howard J., Murashov V.V., Lowe B.D., Lu M.-L. (2020). Industrial exoskeletons: Need for intervention effectiveness research. Am. J. Ind. Med..

[130] Pillai M.V., Van Engelhoven L., Kazerooni H. (2020). Evaluation of a Lower Leg Support Exoskeleton on Floor and Below Hip Height Panel Work. Hum. Factors.

[131] Neubauer B., Durfee W. (2016). Preliminary Design and Engineering Evaluation of a Hydraulic Ankle–Foot Orthosis. J. Med. Devices.

[132] Antwi-Afari M.F., Li H., Anwer S., Li D., Yu Y., Mi H.-Y., Wuni I.Y. (2021). Assessment of a passive exoskeleton system on spinal biomechanics and subjective responses during manual repetitive handling tasks among construction workers. Saf. Sci..

[133] Kapeller A., Felzmann H., Fosch-Villaronga E., Hughes A.-M. (2020). A Taxonomy of Ethical, Legal and Social Implications of Wearable Robots: An Expert Perspective. Sci. Eng. Ethics.

[134] Maurice P., Allienne L., Malaise A., Ivaldi S. Ethical and Social Considerations for the Introduction of Human-Centered Technologies at Work. Proceedings of the IEEE Workshop on Advanced Robotics and its Social Impacts (ARSO).

[135] Theurel J., Desbrosses K., Roux T., Savescu A. (2018). Physiological consequences of using an upper limb exoskeleton during manual handling tasks. Appl. Ergon..

[136] Nnaji C., Okpala I., Gambatese J., Jin Z. (2023). Controlling safety and health challenges intrinsic in exoskeleton use in construction. Saf. Sci..

[137] Kapeller A., Felzmann H., Fosch Villaronga E., Nizamis K., Hughes A.M. (2021). Implementing Ethical, Legal, and Societal Considerations in Wearable Robot Design. Appl. Sci..

[138] Cai M., Ji Z., Li Q., Luo X. (2023). Safety evaluation of human–robot collaboration for industrial exoskeleton. Saf. Sci..

[139] Xia J., Durfee W.K. (2013). Analysis of Small-Scale Hydraulic Actuation Systems. J. Mech. Des..

[140] Diller S., Majidi C., Collins S.H. A lightweight, low-power electroadhesive clutch and spring for exoskeleton actuation. Proceedings of the 2016 IEEE International Conference on Robotics and Automation (ICRA).

[141] Aliman N., Ramli R., Amiri M.S. (2024). Actuators and transmission mechanisms in rehabilitation lower limb exoskeletons: A review. Biomed. Eng./Biomed. Tech..

[142] Redlarski G., Blecharz K., Dąbkowski M., Pałkowski A., Tojza P.M. (2012). Comparative analysis of exoskeletal actuators. Pomiary Autom. Robot..

[143] Deshpande A., Hingwe A., Bae J.H., Naquila G., Zhang H. (2024). Novel bio-inspired soft actuators for upper-limb exoskeletons: Design, fabrication and feasibility study. Front. Robot. AI.

